# Hemorragia cerebral fatal después de una mordedura de serpiente *Bothrops asper* en la región del Catatumbo, Colombia

**DOI:** 10.7705/biomedica.5181

**Published:** 2020-12-09

**Authors:** Jairo Lizarazo, Ramón Patiño, Diego Lizarazo, Guadalupe Osorio

**Affiliations:** 1 Departamento de Medicina Interna, Hospital Universitario Erasmo Meoz, Universidad de Pamplona, Cúcuta, Colombia Universidad de Pamplona Departamento de Medicina Interna Universidad de Pamplona Cúcuta Colombia; 2 Departamento de Cirugía, Hospital Universitario Erasmo Meoz, Universidad de Pamplona, Cúcuta, Colombia Universidad de Pamplona Departamento de Cirugía Universidad de Pamplona Cúcuta Colombia

**Keywords:** Bothrops, venenos de víboras, mordeduras de serpientes, hemorragia cerebral, Colombia, Bothrops, viper venoms, snake bites, intracranial hemorrhage, Colombia

## Abstract

Las mordeduras de serpientes son un problema de salud pública en regiones tropicales y subtropicales del mundo. Ocurren, especialmente, en trabajadores rurales, y son una importante fuente de discapacidad y mortalidad.

Se presenta el caso de un hombre de 59 años, agricultor de la región del Catatumbo (Colombia), quien sufrió la mordedura de una serpiente *Bothrops asper,* la cual le produjo una hemorragia cerebral fatal.

Se llama la atención sobre el grave trastorno hemorrágico en contraste con los leves cambios en el sitio de la mordedura, así como sobre la necesidad del tratamiento temprano de la intoxicación con el suero antiofídico, incluso, en ausencia de manifestaciones cutáneas significativas.

## Abstract (en)

Snake bites are a public health problem in tropical and subtropical regions of the world. They occur especially in rural workers and are an important source of disability and mortality.

We present the case of a 59-year-old farmer from the Catatumbo region of Colombia who was bitten by a *B. asper* snake and suffered a fatal brain hemorrhage after the event. We draw attention to the severe bleeding disorder in contrast with the slight changes at the site of the bite, as well as on the importance of the early treatment of poisoning with antivenom even in the absence of significant skin manifestations.

Las mordeduras de serpientes son un problema de salud pública desatendido en muchos países tropicales y subtropicales. Se calcula que cada año cerca de 2,7 millones de personas sufren envenenamiento debido a estas mordeduras, y que, aproximadamente, 100.000 mueren y 400.000 quedan discapacitadas de forma permanente ^1^. En Latinoamérica, la incidencia total de envenenamiento por mordedura de serpiente oscila entre 5 y 62 casos por 100.000 personas al año ^2^. En Colombia, se notificaron 5.286 casos de accidente ofídico en el 2018, de los cuales 385 (7,3 %) ocurrieron en Norte de Santander ^3^. La mayoría de estos accidentes son causados por serpientes del género *Bothrops,* con una letalidad del 0,7 % ^4^.

Los signos clínicos más comunes de la mordedura de serpientes del género *Bothrops* son eritema, dolor y edema en el sitio de mordedura. Sin embargo, en los casos graves pueden presentarse hemorragias en distintos órganos, entre ellos el cerebro ^5^. Se presenta un caso de hemorragia cerebral fatal en un adulto luego de la mordedura por *Bothrops asper.*

## Presentación del caso

Se trata de un agricultor de 59 años procedente del área rural del corregimiento de La Gabarra, municipio de Tibú, en la región del Catatumbo colombiano, quien consultó por un cuadro clínico de 12 horas de evolución caracterizado por cefalea global intensa, sangrado en las encías y hematuria. Tres días antes había sido mordido en el pulgar derecho por una serpiente *B. asper* identificada con base en la descripción dada por el paciente y un familiar. En el Centro de Salud de La Gabarra fue valorado y le aplicaron tres viales de suero antiofídico polivalente del Instituto Nacional de Salud.

Fue remitido al Hospital Regional del Norte de Tibú, en donde ingresó somnoliento, pero orientado y con signos vitales normales. Se determinaron los tiempos de coagulación como prolongados (tiempo de protrombina: 50 segundos, tiempo de tromboplastina: 54 segundos), por lo cual se decidió aplicar otros tres viales de suero antiofídico. En las siguientes horas, el paciente presentó deterioro del estado de conciencia hasta entrar en coma, con dilatación y falta de reacción de la pupila derecha, y sangre no coagulable.

Se le aplicaron otros cuatro viales de suero antiofídico y fue remitido al Hospital Universitario Erasmo Meoz de Cúcuta, donde ingresó el 11 de marzo de 2019, en coma y con los siguientes signos vitales: tensión arterial, 126/75 mm Hg; frecuencia cardiaca, 82 latidos por minuto; frecuencia respiratoria de 18 por minuto; temperatura de 36 °C; puntaje de 6/15 en la escala de coma de Glasgow, y anisocoria por midriasis paralítica derecha (pupila derecha, 5 mm y pupila izquierda, 3 mm) con reacción de decorticación.

**Figura 1:**
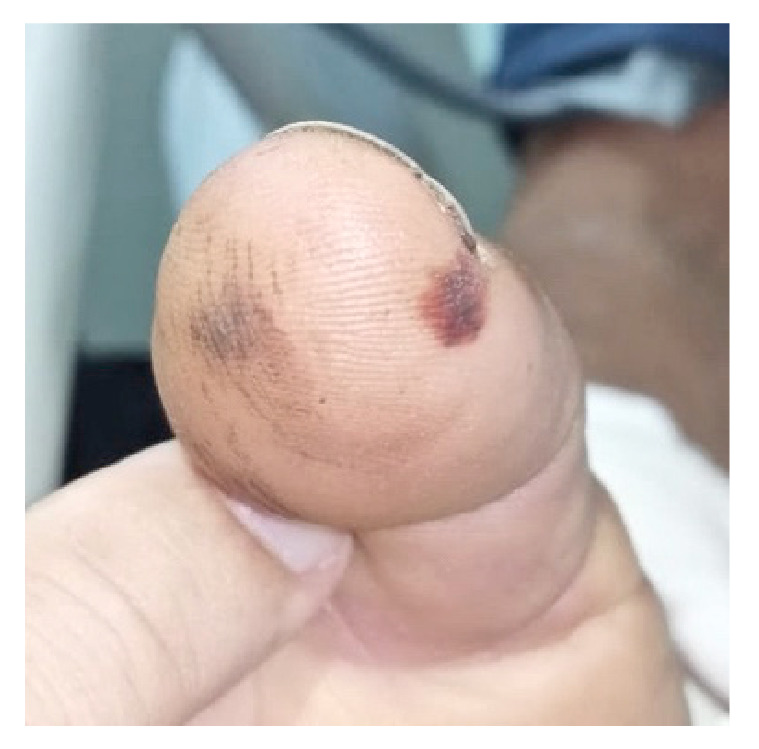
Dos equimosis por la mordedura de la serpiente en la falange distal del pulgar derecho

**Figura 2:**
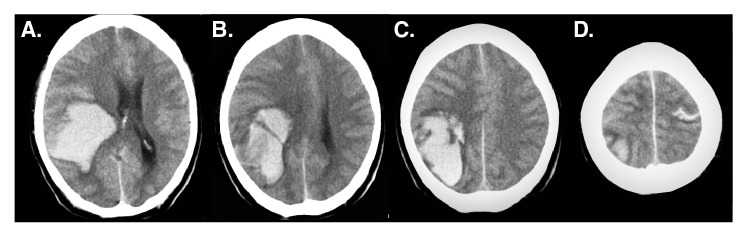
Tomografía axial simple de cráneo: **a, b, c.** Hemorragia cerebral parietal derecha extensa y hemorragia de núcleos basales derechos con desvío de la línea media; **d.** Hemorragia subaracnoidea cortical parietal izquierda

Se observaron dos lesiones por mordedura de serpiente en la falange distal del pulgar derecho, con signos de sangrado y leve edema de la mano derecha (figura 1). En la tomografía computarizada simple de cráneo, se observó una gran hemorragia cerebral témporo-parietal y de los núcleos basales derechos que ejercía efecto de masa al comprimir el atrio ventricular y desviar la línea media, y cuyo volumen se calculó en 97 ml; había pequeñas hemorragias subaracnoideas corticales parietales izquierdas y en la cisterna *ambiens* izquierda. Además, se observó edema cerebral y del tallo cerebral (figura 2).

Inmediatamente, se hizo la intubación orotraquial y se dio asistencia respiratoria mecánica. Las nuevas pruebas de coagulación fueron normales, pero se observó una leve hipofibrinogenemia. Los resultados de los exámenes paraclínicos están consignados en el cuadro 1. Ese mismo día, el paciente fue sometido a craneotomía y drenaje del hematoma cerebral y, posteriormente, se le trasladó a la unidad de cuidados intensivos. Su evolución no fue satisfactoria y presentó una falla multiorgánica que desembocó en su muerte el 16 de marzo de 2019, nueve días después del accidente ofídico.

**Cuadro 1:** Resultados de los exámenes paraclínicos del ingreso

Cuadro hemático	
Hemoglobina (g/dl)	12,4
Leucocitos (por µl)	13.270
Polimorfonucleares (por µl)	11.450
Plaquetas (por µl)	231.000
Pruebas de coagulación	
Tiempo de protrombina (s), (control: 11,9 s)	12,60
INR	1,06
Tiempo parcial de tromboplastina (s), (control: 29 s)	22,90
Fibrinógeno (mg/dl), (normal: 200-400 mg/dl)	139,90
Química sanguínea	
Nitrógeno ureico (mg/dl)	20
Creatinina (mg/dl)	0,64
Electrolitos séricos	
Sodio (mmol/L)	140
Potasio (mmol/L)	3,50
Parcial de orina	Normal
Electrocardiograma	Bradicardia sinusal
Radiografía del tórax	Normal

### Consideraciones éticas

La publicación de este caso fue autorizada por el Comité de Ética de Investigación del Hospital Universitario Erasmo Meoz de Cúcuta.

## Discusión

Los accidentes ofídicos han sido motivo de estudio en Colombia y son numerosas las publicaciones sobre el tema ^6^^-^^13^. También se han descrito las complicaciones hemorrágicas ^9^^,^^10^ e isquémicas ^14^^,^^15^ del sistema nervioso central, especialmente las asociadas con la mordedura de *B. asper.*

*Bothrops asper* es responsable de la mayoría (50-80 %) de las mordeduras de serpientes en Centroamérica y en las regiones del norte de Suramérica ^2^. Las mordeduras afectan principalmente a hombres del área rural atacados durante sus actividades laborales ^16^.

Este paciente provenía del Catatumbo, una región con una extensión de 10.089 km^2^, ubicada en el departamento de Norte de Santander, al nororiente de Colombia, en la frontera con la República Bolivariana de Venezuela. Es un territorio formado por zonas montañosas y planas, con gran biodiversidad y riqueza hídrica. Está conformada por 11 municipios y tiene una población de 250.000 habitantes, la mitad de ellos residentes en las áreas rurales, entre las cuales se cuentan los resguardos de la comunidad indígena barí. Es una región marginada, con poca presencia estatal y necesidades básicas insatisfechas para la mayoría de la población. Además, ha sido escenario de conflictos armados y del accionar del narcotráfico dado el desarrollo de una economía cocalera ^17^.

Las manifestaciones clínicas producidas por el veneno de *B. asper* pueden ser locales o sistémicas y afectan tres sistemas: el musculoesquelético, el hematológico y el renal. Las principales manifestaciones son: edema local, equimosis, ampollas, dermonecrosis y mionecrosis, disfibrinogenemia, trombocitopenia, sangrado sistémico, hipotensión y alteraciones renales. Además, se puede observar infección de tejidos blandos, falla renal aguda, síndrome compartimental, hemorragia del sistema nervioso central y, en mujeres embarazadas, aborto, pérdidas fetales y desprendimiento de la placenta ^2^. En el presente caso, las manifestaciones locales fueron leves: solo edema y equimosis.

La hemorragia del sistema nervioso central ocurre en 2 a 3 % de las víctimas de la mordedura de *B. asper* y es una de las complicaciones más graves. Las hemorragias pueden ser cerebrales, principalmente lobares, ventriculares, medulares, subaracnoideas, cerebelosas, subdurales o epidurales. Los síntomas dependen de la localización del sangrado y, cuando este es masivo, predomina la hipertensión intracraneal ^2^^,^^18^. En el presente caso, una hemorragia cerebral masiva produjo hipertensión intracraneal y herniación del lóbulo temporal con compresión del mesencéfalo, así como hemorragia subaracnoidea, situación que empeoró el pronóstico neurológico (figura 2).

En pacientes mordidos por serpientes del género *Bothrops,* se han descrito con menor frecuencia los infartos encefálicos, la gran mayoría cerebrales corticales; ocasionalmente, afectan varios territorios arteriales, pero pocas veces hay transformación hemorrágica. La causa del infarto isquémico es controvertida. Se han propuesto algunas teorías sobre su etiopatogenia, entre ellas, la presencia de toxinas del veneno como causantes de hipercoagulabilidad y de daño endotelial, vasculitis inmunológica e hipotensión sistémica ^18^.

El veneno de *B. asper* contiene proteínas que pertenecen, por los menos, a ocho familias: metaloproteinasas, proteasas de serina, proteína similar a la lectina de tipo C, L-aminoácido oxidasa, desintegrina, fragmento DC, proteína secretora rica en cisteína y fosfolipasa A2 (PLA2). En este veneno se han aislado y caracterizado unas 25 proteínas pertenecientes a estas familias ^19^.

La hemorragia sistémica y la coagulopatía constituyen los dos efectos sistémicos más reconocidos del envenenamiento por mordedura de *B. asper,* tanto clínica como experimentalmente. Las metaloproteinasas del veneno de serpiente P-III (especialmente la batroxrhagina), las proteinasas de serina y la proteína similar a la lectina de tipo C, son responsables de la hemorragia sistémica, la disfibrinogenemia, la trombocitopenia y la disminución de la agregación plaquetaria ^20^^,^^21^.

Las alteraciones cardiovasculares que llevan al choque son el resultado de los efectos combinados de las toxinas hemorrágicas, de otros componentes del veneno, como la PLA2 y las aminas biógenas, que aumentan la permeabilidad vascular responsable de la hipotensión, así como del efecto cardiotóxico de la miotoxina PLA2. Las alteraciones renales probablemente se deben a la acción citotóxica directa de las nefrotoxinas, y a la isquemia renal debida a la hipovolemia y la hipoperfusión tisular. Sin embargo, la patogenia de las alteraciones cardiovasculares y de la falla renal no se ha dilucidado del todo, a pesar de los conocimientos adquiridos sobre otros venenos de serpientes del género *Bothrops*^20^^,^^21^.

Las alteraciones más frecuentes de los exámenes de laboratorio son la disminución del fibrinógeno, la cual ocurre en el 60 al 70 % de los casos de forma precoz, usualmente de los 30 a los 60 minutos del envenenamiento, y la trombocitopenia, que ocurre del 15 al 30 % de los casos. En los envenenamientos moderados y graves, se observa alteración de las pruebas de coagulación (tiempo de protrombina, tiempo parcial de tromboplastina, dímero D y productos de degradación del fibrinógeno o la fibrina). En el seguimiento, estas pruebas se deben practicar frecuentemente ^2^.

Una manera rápida, sencilla y eficaz para detectar el consumo de fibrinógeno es hacer la prueba de coagulación de sangre total a los 20 minutos (se puede complementar con la medición a los 30 minutos), la cual indica coagulopatía por consumo cuando no se forma coágulo en el tiempo estipulado ^22^^,^^23^. Esta prueba es útil en la valoración inicial del paciente con mordedura de serpiente en sitios en donde no se dispone de un laboratorio; se hace a la cabecera del paciente y su sensibilidad y especificidad son aceptables ^23^. Una prueba positiva significa que se han inoculado unos 300 mg de veneno. La presencia de sangre no coagulable y la de trombocitopenia en el momento del ingreso hospitalario, son dos factores que se asocian con el sangrado sistémico ^21^.

En Colombia, la guía oficial recomienda que, en casos de envenenamiento grave, se apliquen 10 a 12 ampollas del suero polivalente manufacturado por el Instituto Nacional de Salud, el cual tiene la potencia de neutralizar 70 mg de veneno de *B. asper* por cada ampolla de 10 ml ^2^.

La mortalidad asociada con los eventos cerebrovasculares causados por el envenenamiento de serpientes del género *Bothrops* es del 62 % y los supervivientes usualmente quedan con secuelas ^24^. En un estudio brasileño reciente ^16^, se encontró que una demora de más de seis horas en la administración del antiveneno y la edad de 65 o más años, se asocian en forma independiente con la mortalidad en los pacientes que sufren intoxicación por veneno de serpiente. En el mismo sentido, una tardanza de seis o más horas para dar inicio al tratamiento específico se ha asociado significativamente con la gravedad de la intoxicación ^8^^,^^24^. Se ha comprobado que la hemorragia del sistema nervioso central también está relacionada con la demora en iniciar el tratamiento específico, lo que sucedió en este paciente ^10^^,^^25^.

Este caso llama la atención por los leves signos locales de envenenamiento que contrastan con el grave sangrado del sistema nervioso central. Probablemente, los pocos cambios locales influyeron para que el paciente tardara en consultar. Hay que tener en cuenta que las afectaciones locales leves no excluyen el consumo masivo de fibrinógeno y la presencia de hemorragia sistémica. Este es un hecho conocido y se ha postulado que ocurre cuando la mordedura es por una serpiente joven. Se han demostrado diferencias en la composición de los venenos según la edad de las serpientes *B. asper.* Gutiérrez, *et al.*^26^, demostraron, mediante estudios electroforéticos y de inmunoelectroforesis, que el veneno de las serpientes recién nacidas es más hemorrágico y el de las serpientes adultas tiende a producir más mionecrosis.

Desde el punto de vista ontogenético, en los estudios de proteómica se ha determinado el predominio de las metaloproteínasas de clase PIII (las toxinas hemorrágicas más potentes) en las serpientes jóvenes, en tanto que, en las adultas, predominan las metaloproteinasas de clase PI (inducen hemorragia local); además, las serpientes adultas tienen un conjunto diferente de fosfolipasas A2 (inductoras más de dermonecrosis y mionecrosis) ^27^^,^^28^.

Los accidentes ofídicos graves, como el que se presenta aquí, revelan que este problema de salud está desatendido e invitan a coordinar esfuerzos globales para disminuir su impacto en la mortalidad y la discapacidad ^1^. Asimismo, obligan al personal sanitario a cargo de estos pacientes en zonas remotas (como la región del Catatumbo nortesantandereano), a mejorar el conocimiento de esta condición y el cumplimiento de las guías de manejo, y a garantizar el adecuado abastecimiento del suero antiofídico.
